# Unraveling the function of TSC1-TSC2 complex: implications for stem cell fate

**DOI:** 10.1186/s13287-025-04170-3

**Published:** 2025-02-04

**Authors:** Shuang Wang, Ruishuang Ma, Chong Gao, Yu-Nong Tian, Rong-Gui Hu, Han Zhang, Lan Li, Yue Li

**Affiliations:** 1 Institute of Traditional Chinese Medicine, Tianjin University of Traditional Chinese Medicine, Tianjin, China; 2 School of Medicine, Institute of Brain and Cognitive Science, Hangzhou City University, Hangzhou, Zhejiang China; 3 State Key Laboratory of Brain-Machine Intelligence, Liangzhu Laboratory, School of Medicine, Zhejiang University, Zhejiang, China; 4 State Key Laboratory of Quality Research in Chinese Medicine, Institute of Chinese Medical Sciences, University of Macau, Macau, China; 5 Department of Pharmaceutical Sciences, Faculty of Health Sciences, University of Macau, Macau, China

**Keywords:** Tuberous sclerosis complex, Mammalian target of rapamycin, Stem cell

## Abstract

**Background:**

Tuberous sclerosis complex is a genetic disorder caused by mutations in the *TSC1* or *TSC2* genes, affecting multiple systems. These genes produce proteins that regulate mTORC1 activity, essential for cell function and metabolism. While mTOR inhibitors have advanced treatment, maintaining long-term therapeutic success is still challenging. For over 20 years, significant progress has linked *TSC1* or *TSC2* gene mutations in stem cells to tuberous sclerosis complex symptoms.

**Methods:**

A comprehensive review was conducted using databases like Web of Science, Google Scholar, PubMed, and Science Direct, with search terms such as “tuberous sclerosis complex,” “TSC1,” “TSC2,” “stem cell,” “proliferation,” and “differentiation.” Relevant literature was thoroughly analyzed and summarized to present an updated analysis of the TSC1-TSC2 complex’s role in stem cell fate determination and its implications for tuberous sclerosis complex.

**Results:**

The TSC1-TSC2 complex plays a crucial role in various stem cells, such as neural, germline, nephron progenitor, intestinal, hematopoietic, and mesenchymal stem/stromal cells, primarily through the mTOR signaling pathway.

**Conclusions:**

This review aims shed light on the role of the TSC1-TSC2 complex in stem cell fate, its impact on health and disease, and potential new treatments for tuberous sclerosis complex.

**Graphical abstract:**

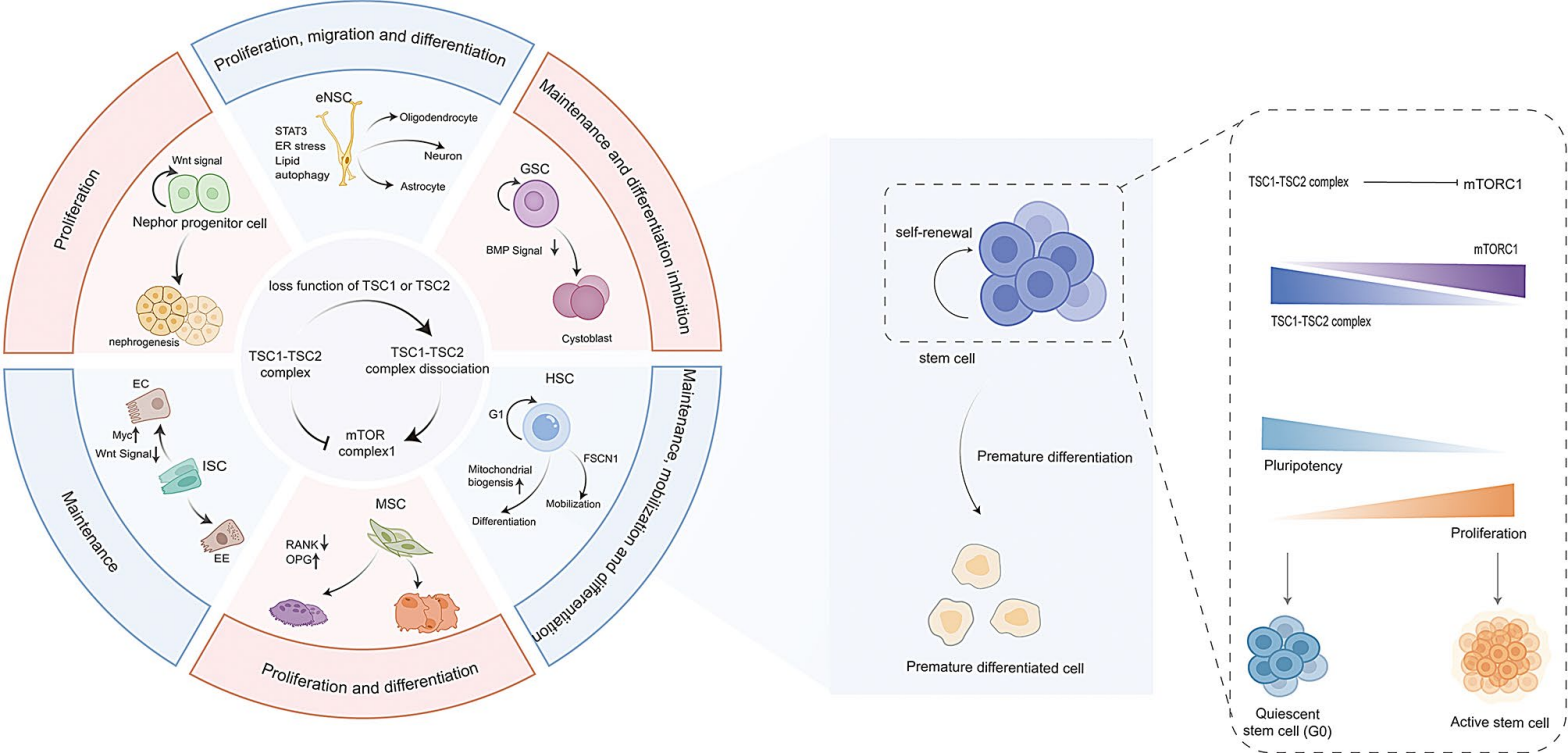

## Introduction

Tuberous sclerosis complex is a genetic disorder characterized by hamartomas in organs, like the brain, heart, skin, eyes, kidney, lung, and liver [1–2]. It mainly presents with skin lesions and neurological symptoms [1], such as epilepsy, intellectual disability, autism spectrum disorders (ASD), and cognitive impairment [3]. It affects about 1 in 20,000 to 100,000 newborns [4–5]. Tuberous sclerosis complex results from loss-of-function mutations in the *TSC1* or *TSC2* genes, which encode proteins crucial for tumor suppression [6–7]. Nearly one-third of cases are inherited, while two-thirds occur spontaneously [5]. The TSC1-TSC2 complex, along with the auxiliary subunit TBC1D7, inhibits the mammalian target of rapamycin complex 1 (mTORC1), a key serine/threonine protein kinase crucial for cell growth, proliferation, and metabolism [8]. Mutations in *TSC1* or *TSC2* genes disrupt the function of TSC1-TSC2 complex, leading to mTORC1 hyperactivation, which is thought to cause tuberous sclerosis complex [9]. Thus, inhibiting mTORC1 is a potential treatment strategy, with rapamycin (sirolimus), an mTORC1 inhibitor, used clinically. However, rapamycin treatment carries significant risks, potential side effects and short-term impacts. Furthermore, clinical trials have demonstrated that rapamycin does not ameliorate neuropsychiatric disorders associated with tuberous sclerosis complex [3, 8, 10–12]. Understanding the molecular and cellular mechanisms of the TSC1-TSC2 complex could improve disease management strategies [4].

Stem cells play a crucial role in tissue development, homeostasis and regeneration, owing to their ability to self-renew and differentiate [13–14]. Recent studies have uncovered that the disruption of the TSC1-TSC2 complex in stem cells is linked to tuberous sclerosis complex, highlighting its importance in regulating stem and progenitor cell functions. This review begins with a brief overview of the structures and roles of TSC1, TSC2, and TBC1D7 within the complex. We emphasize the importance to regulate the TSC1-TSC2 complex in various stem cells and identify key research areas that could aid in developing therapies for tuberous sclerosis complex -associated diseases.

## TSC1-TSC2 complex

### TSC1, TSC2, and TBC1D7 domain structure and function

The TSC1-TSC2 complex consists of TSC1, TSC2, and TBC1D7 subunits. Mutations in *TSC1* or *TSC2* cause tuberous sclerosis complex, but no germline mutations in TBC1D7 have been found in patients [15].

**Fig. 1:**
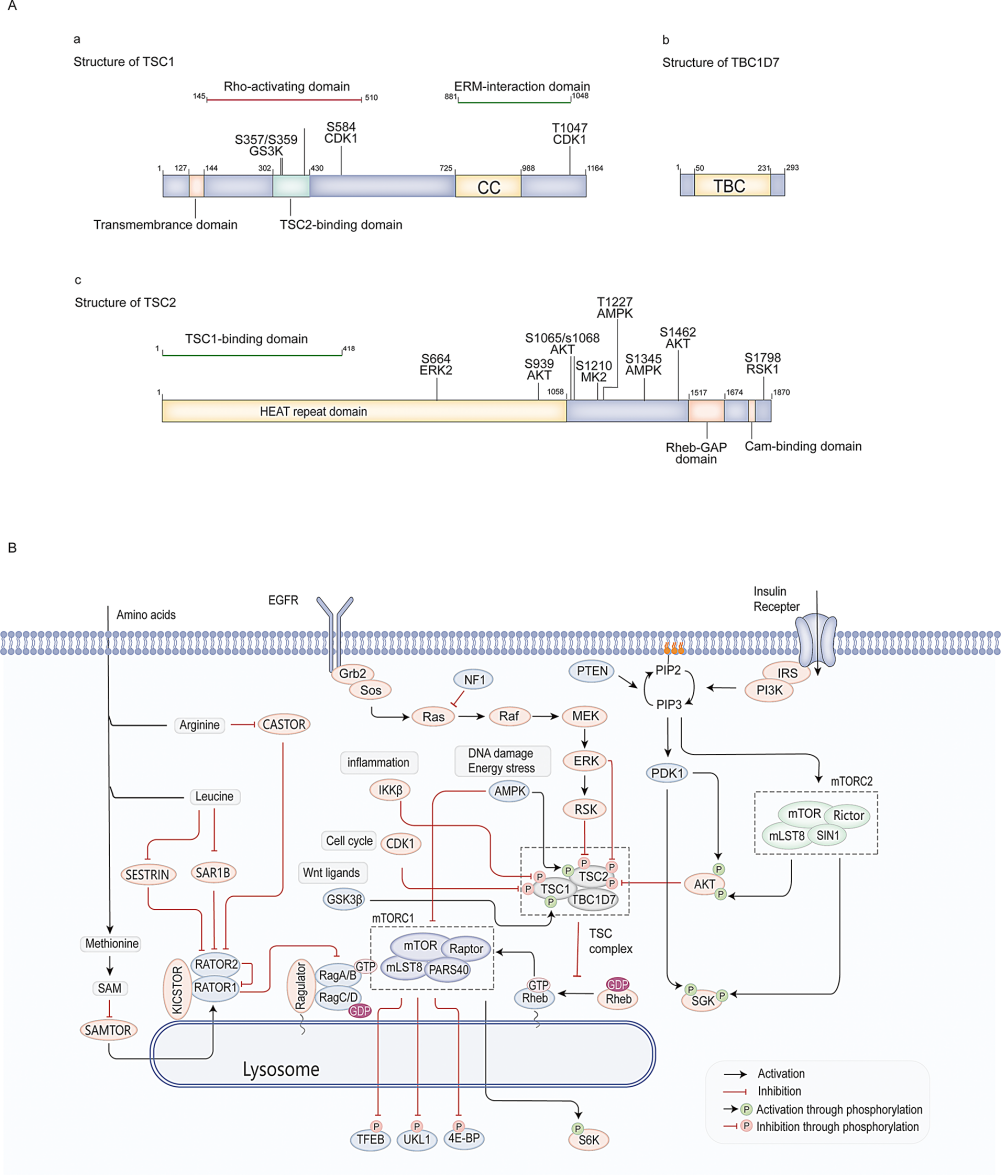
Basic structure and biology function of TSC1 (hamartin), TSC2 (tuberin) and TSC1D7. **(A).** Structure of TSC1 (hamartin), TSC2 (tuberin) and TSC1D7. Only inhibitory phosphorylation sites are shown. (**a**) Structure of TSC1 and inhibitory phosphorylation sites with GS3K and CDK1. (**b**) Structure of TBC1D7. (**c**) Structure of TSC2 and inhibitory phosphorylation sites with ERK2, AKT MK2, AMPK and RSK1. **(B).** TSC1, TSC2, and TBC1D7 form a TSC1-TSC2 complex that regulates mTORC1. TSC1-TSC2 complex acts as a signal integration to maintain intracellular homeostasis. Only major regulators and targets are shown

The *TSC1* gene, on chromosome 9 (9q34.13) with 23 exons, encodes a 130 kDa protein present in various tissues (Tables 1 and 2), featuring a transmembrane domain and coiled-coil domains (CCs, residues 746–971) (Fig. 1A) [16]. Two TSC1 proteins form the complex by aligning their coiled-coil domains in parallel to create an arch structure that binds with TSC2. The C-terminal tip of TBC1D7 links two TSC1 molecules by interacting with CCs [17–19]. The Ezrin-radixin-moesin (ERM) domain of TSC1 (residues 881–1084) controls actin dynamics and cell adhesion, inhibiting tumor growth (Fig. 1A). Additionally, TSC1 residues 145–510 activate Rho GTPase, promoting cell-matrix focal adhesions and actin stress fibers (Fig. 1A) [20]. Neurofilament-light chain (NF-L), which stabilizes neurons and regulates axonal growth, binds with TSC1 protein in primary cortical neurons, possibly influencing neuronal cytoskeleton formation for normal neural development and function [21].

**Table 1:** Characteristics of TSC1 and TSC2

Items	TSC1	TSC2	References
Chromosomal location	9q34	6p13.3	[6, 98]
Size	55 kb	40 kb	[6, 98]
Number of exons	23	41	[6, 98]
Mutation occurrence	10-15% of sporadic cases	75-80% of sporadic cases	[4, 99]
Prevailing mutations	mostly nonsense mutations and small deletions, lack of vulnerable point	mostly missense mutations or deletions, lack of vulnerable point	[95]
Phenotype	Less severe	More severe	[5]
Protein	Hamartin (TSC1)	Tuberin (TSC2)	[6, 98]
Protein size	1164 amino acids, 130 kDa	1807 amino acids, 200 kDa	[6, 98]
Protein function	Bind with TSC2 and TBC1D7, and hamartin regulates cell adhesion	Bind with TSC1and TBC1D7, GTPase active, TSC2 regulates cell cycle,	[15, 20, 100–101]

**Table 2:** The role of TSC1 and TSC2 in regulating stem cell behaviors

Protein	Cell type	Species	Effects	Mechanisms	References
TSC1	eNPCs	Mouse	Maintenance	mTORC1	[58]
TSC2	NSCs	Human	Proliferation	mTORC1, PAX6	[102]
TSC1	eNPCs	Mouse	Migration and neuronal differentiation	mTOR- S6K	[103]
TSC1	eNSCs	Mouse	Maintenance and differentiation inhibition	mTORC1, STAT3	[57]
TSC1	GSCs	Drosophila	Maintenance and differentiation inhibition	mTORC1-S6K	[69]
TSC2	GSCs	Drosophila	Maintenance and differentiation inhibition	mTORC1-S6K	[69]
TSC2	SPCs	Mouse	Heterogeneity and differentiation	mTORC1	[70]
TSC1	NPCs	Mouse	Migration		[62]
TSC1	NPCs	Mouse	Migration	mTORC1 TFEB	[63]
TSC1	Nephron progenitor cells	Mouse	Proliferation	Raptor	[74]
TSC1	Nephron progenitor cells	Mouse	Proliferation	mTORC1	[75]
TSC1	Nephron progenitor cells	Mouse	Proliferation	Wnt signal	[73]
TSC1	ISC	Drosophila	Maintenance and differentiation	mTORC1-S6K	[104]
TSC2	ISC	Drosophila	Maintenance and differentiation	mTORC1	[80]
TSC2	ISC	Mouse	Maintenance	mTORC1, FZD	[79]
TSC1	HSCs	Mouse	Maintenance	ROS production	[83]
TSC1	HSCs	Mouse	Mobilization, proliferation and differentiation.	mTORC1	[82]
TSC1	MSCs	Mouse	Proliferation and differentiation	mTORC1	[90]
TSC1	BM-MSC	Mouse	Maintenance and proliferation	mTORC1	[91]

The TSC2 gene on chromosome 16 (16p13.3) has 44 exons and encodes the 200 kDa TSC2 protein (tuberin), a key functional constituent of the TSC1-TSC2 complex (Table 1) [4]. TSC2 comprises a HEAT repeat domain with 18 HEAT domains, a dimerization domain (DD), and a C-terminal GTPase-activating protein (GAP) catalytic domain (Fig. 1A). The N-terminal 12 HEAT domains of TSC1 stabilize it by interacting with its CCs. The GAP catalytic domain blocks mTOR activation by converting Rheb-GTP to its inactive GDP form. Additionally, four positively charged regions around the DD and GAP domains might engage with phosphorylated lipids in lysosomal membranes via electrostatic interactions, aiding the TSC1-TSC2 complex in localizing to lysosomes and controlling mTORC1 [17, 22].

TBC1D7, a member of TBC domain family, is the third functional subunit of the TSC1-TSC2 complex [15]. It binds specifically to the C-terminal of TSC1, influencing the complex’s stability and TSC1-TSC2 interaction [15, 17]. TBC domains serve as GAPs for Rab GTPases and cytokinesis-controlling GTPases, suggesting TBC1D7 can regulate Small G Proteins (Fig. 1A). Mutations in the TBC1D7 gene do not cause tuberous sclerosis complex, but its deficiency can lead to symptoms linked to mTOR abnormalities, like intellectual disability and megalocephaly [23–24]. Reducing TBC1D7 protein levels increases mTORC1 signaling less than reducing TSC1 or TSC2 proteins in Hela cells [15], explaining why TBC1D7 mutations alone do not lead to tuberous sclerosis complex.

### Regulation of TSC1-TSC2 complex

The TSC1-TSC2 complex’s function relies on the interaction between TSC1 and TSC2 proteins, with changes to either affecting its activity [25]. Post-translational modifications (PTMs) regulate this complex, primarily within the PI3K/ AKT pathway. Growth factors activate PI3K, leading to PIP3 synthesis and the recruitment of PKD and AKT to the plasma membrane (Fig. 1B) [26]. PKD phosphorylates AKT at Thr305, partially activating it. This activated AKT then phosphorylates TSC2 at specific residues, negatively regulating the TSC1-TSC2 complex (Fig. 1A, B) [27–28]. Meanwhile, ERK and RSK, part of the Ras-Raf-MEK pathway, phosphorylate TSC2 at Ser664 and Ser1798, leading to inactivation of the TSC1-TSC2 complex (Fig. 1A, B) [29]. Additionally, IKKβ phosphorylates TSC1 at Ser487 and Ser511 in response to inflammation, further negatively regulating the complex (Fig. 1A, B) [30]. AMP-activated protein kinase (AMPK) activates the TSC1-TSC2 complex by phosphorylating TSC2 at Thr1227 and Ser1345 during energy stress, suppressing mTORC1(Fig. 1A, B) [31]. The main regulation of the TSC1-TSC2 complex is through phosphorylation of TSC1 and TSC2. Additionally, acetylation of TSC2 by arrest-defective protein 1 (ADR1) helps prevent tumorigenesis [32]. Nevertheless, further research is required to elucidate how different post-translational modifications affect TSC1 and TSC2 protein regulation, which could greatly influence the development of tuberous sclerosis complex treatments.

### Regulation of signaling pathway by TSC1-TSC2 complex

Evidence increasingly suggests that the TSC1-TSC2 complex acts as a central hub, regulating cell growth by integrating signals from growth factors, amino acids, and ATP to activate the mTOR pathway (Fig. 1B) [33]. The main cause of tuberous sclerosis complex is the overactivation of the mTOR pathway due to the inactivation of the TSC1-TSC2 complex [3–4, 8]. In mammals, mTOR forms two distinct complexes, mTOR Complex 1 (mTORC1) and mTOR Complex 2 (mTORC2), each with specific functions [34].

mTORC1, consisting of mTOR, mLST8, and RAPTOR, is crucial for protein synthesis. RAPTOR is vital for mTORC1’s positioning and substrate recruitment (Fig. 1B) [33]. mTORC1 enhances translation initiation by phosphorylating ribosomal S6 kinases (S6K) and eukaryotic initiation factor 4E­binding proteins1 (4E-BP1) (Fig. 1B) [35–36]. The unphosphorylated 4E-BP1 binds to eIF4E, preventing the eIF4E complex assembly and inhibiting 5’ cap-dependent mRNA translation. When mTORC1 phosphorylates 4E-BP1, eIF4E is released, promoting mRNA translation initiation [37]. mTORC1 also enhances mitochondrial gene expression via 4E-BP1 phosphorylation, boosting ATP production for energy metabolism homeostasis [38]. Additionally, mTORC1 phosphorylates S6K1 at Thr389, aiding eIF4B complex formation [37–38]. S6K promotes protein synthesis by degrading PDCD4, an eIF4B inhibitor [37]. Excessive mTORC1 activation, often due to TSC1 or TSC2 gene deficiencies, increases phosphorylated 4E-BP1 and S6K levels, causing abnormal cell growth [11, 39–40]. In skeletal muscle, TSC1 deletion leads to delayed myopathy with autophagic substrates, likely because mTORC1 inhibits autophagy by phosphorylating ULK1(Fig. 1B) [41]. The TSC1-TSC2 complex downregulates mTORC1 to support cell growth and differentiation [38]. The exact location of mTORC1 within cells is crucial for its function [42]. Therefore, studying how the TSC1-TSC2 complex regulates mTORC1’s subcellular activity could provide new insights into treating tuberous sclerosis complex.

The TSC1-TSC2 complex inhibits mTORC1 and activates mTORC2 independently of Rheb [43]. mTORC2 is essential for AKT activation, promoting cell survival, growth, and proliferation by inhibiting substrates like FOXO1/3A, GSK3β, and TSC2 (Fig. 1B) [44–45]. It also phosphorylates SGK1, influencing cellular transport and survival (Fig. 1B) [46]. mTORC2 is negatively regulated by mTORC1 through a feedback loop, while S6K disrupts the PI3K-AKT pathway and attenuate mTORC2 activity by phosphorylating IRS1. AKT mediates the interaction between mTORC1 and mTORC2 by inactivating TSC2. Prolonged mTORC2 activation in postnatal neural stem cells (pNSCs) leads to subependymal nodules (SENs) and subependymal giant cell astrocytomas (SEGAs), key neurological manifestation in tuberous sclerosis complex [47]. Thus, Understanding the TSC1-TSC2 and mTORC2 link may provide new treatments for SEANs.

## Regulation of stem cell behaviors by TSC1-TSC2 complex

Stem cells uniquely self-renew and differentiate, unlike other somatic cells [14]. While mature organs mainly contain differentiated somatic cells for physiological functions, stem cells are crucial for tissue maintenance and development by producing specialized cells based on signals from their microenvironment [13, 48–49]. The mTOR pathway influences stem cell self-renewal and differentiation by managing protein synthesis and ribosome production [50]. Recent studies emphasize the crucial role of the TSC1-TSC2 complex in the mTOR pathway for regulating stem cell fate. Although research has focused on the TSC1/2-mTOR pathways, the effects of lacking TSC1 or TSC2 genes may affect various stem cell types beyond this pathway. Thus, it’s crucial to explore how the TSC1-TSC2 complex specifically regulates stem cells and their lineage functions.

### TSC1-TSC2 complex and neural stem cells

#### Embryonic neural stem cells

**Fig. 2:**
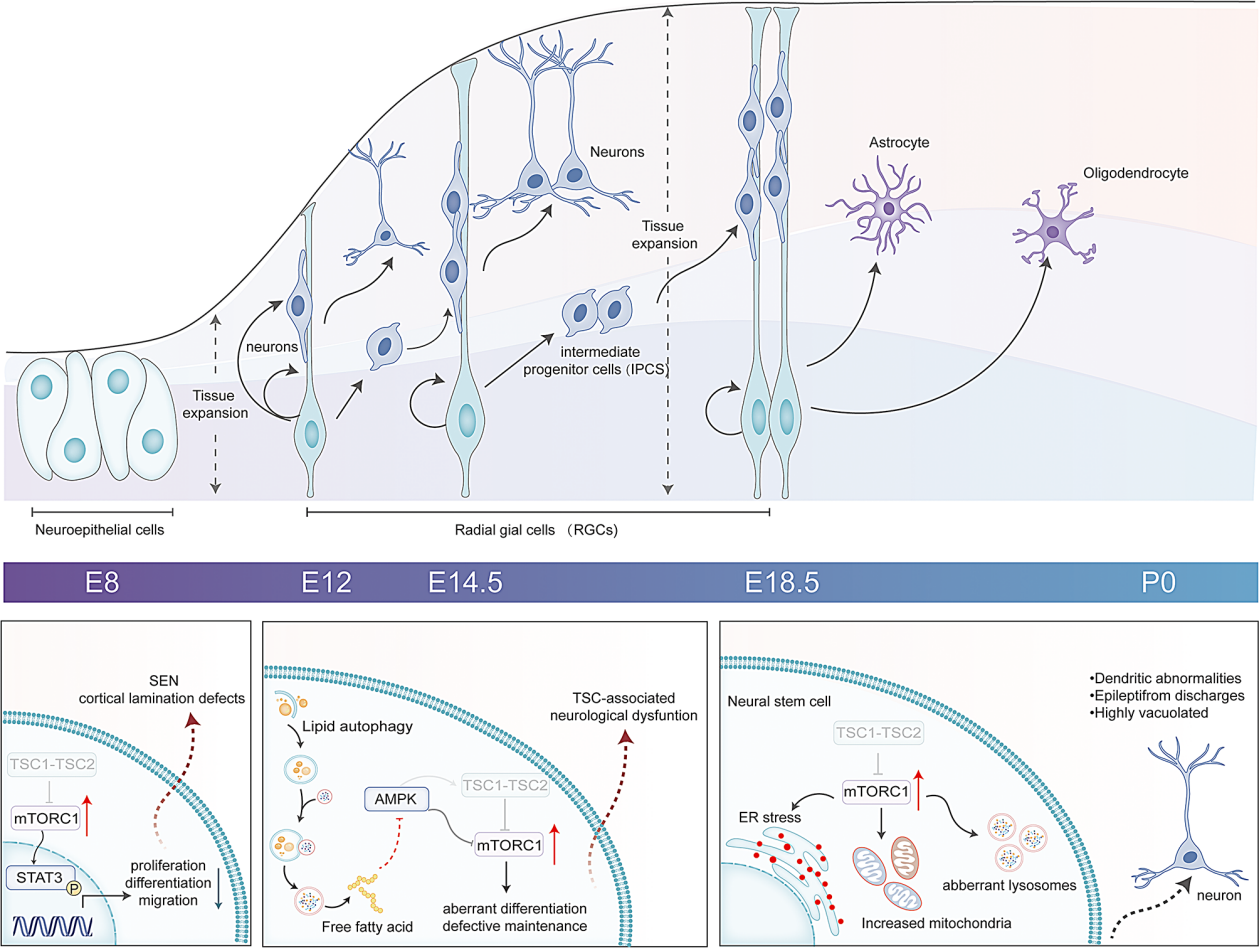
TSC1-TSC2 complex in rodent eNSCs. Knockout *TSC1* or *TSC2* genes in different developmental stages causes corresponding TSC-associated neurological manifestations

Neurogenesis, the process by which neural stem cells (NSCs) give rise to new neurons, occurs throughout life in mammals [51]. In embryos, it is crucial for forming neural networks in the central nervous system (CNS), including various brain regions and the spinal cord [52]. In mice, this process starts at embryonic day 8 (E8) and peaks at day 14 (E14), with neuroepithelial cells losing epithelial characteristics between days 10 (E10) and 12 (E12) (Fig. 2) [52–54]. Neuroepithelial cells transform into radial glial cells (RGCs), which are primary NSCs that differentiate into neurons, sometimes through intermediate progenitor cells (IPCs)(Fig. 2) [51]. These embryonic NSCs can also differentiate into astrocytes and oligodendrocytes later in development [51–52]. The fate of eNSCs, crucial for brain development, is guided by signals at key developmental stages [55]. Mice lacking the *TSC2* gene in radial glial progenitor cells show major embryonic neurogenesis defects, including excessive progenitor cell proliferation, more astrocytes, and enlarged, abnormal neurons [56]. Telencephalic eNSCs with Emx1 expression lacking *TSC1* or *TSC2* genes exhibit impaired cortical development due to early differentiation of neurons and astroglial cells, reducing self-renewal. This is mainly due to abnormal proliferation and differentiation in TSC1-deficient NSCs, driven by mTORC1-dependent AKT inhibition and STAT3 activation (Fig. 2) [57]. Inducing TSC1 deficiency in embryonic neural progenitors (eNPCs) at E13 or E16 leads to vacuolated giant cells. These cells exhibit organelle dysfunction, including increased mitochondria, abnormal lysosomes, heightened stress, and atypical neuron differentiation, leading to epileptogenesis (Fig. 2) [58]. This indicates that TSC1 and TSC2 proteins, and their complex, are crucial for eNSC fate and proper brain development.

#### Adult neural stem cells

Compared to embryonic neurogenesis, adult neurogenesis plays a more restricted role [59]. In mammals, it primarily occurs in two brain areas: the subgranular zone (SGZ) in the hippocampus, where it aids memory formation [60], and the subventricular zone (SVZ), where it supports interneuron development in the olfactory bulb (OB) [61]. Conditional deletion of the *TSC1* gene in aNSCs from the SVZ results in defects in neurogenesis, including abnormal growth of neural progenitors and atypical neuroblast migration, which likely lead to subventricular nodules [62]. Hyperactivation of mTORC1 prevents transcription factor EB (TFEB) from activating, which severely hinders the migration of TSC1-deficient NSPC [63]. Research on the TSC1-TSC2 complex in aNSCs has primarily targeted the SVZ because of brain nodules linked to cognitive deficits in tuberous sclerosis patients. Since adult hippocampal neurogenesis is crucial for memory and cognition, studying the TSC1-TSC2 complex’s impact on aNSCs in the SGZ may yield valuable insights.

In summary, studies from embryonic to adult stages show the TSC1-TSC2 complex is vital for NSC fate determinations. Notably, 80-90% of individuals with tuberous sclerosis complex have cortical tubers, the main cause of epilepsy and mental disability in these patients. Deletion of TSC1 gene in mouse embryonic telencephalic NSCs leads to excessive cell growth and impaired neuronal differentiation, contributing to CT formation [57, 64]. In 2022, a subsequent study using single-cell transcriptomics on induced pluripotent stem cell (iPSC)-derived cerebral organoids from tuberous sclerosis patients revealed that CLIP fate dysregulation is crucial in cortical tuber development [65]. Structural abnormalities similar to subependymal nodules (SEN) and subependymal giant cell astrocytomas (SEGA) occur in mice with TSC1 knockdown in NSCs at various developmental stages. At embryonic day 12.5, a TSC2 mutation in radial glial cells leads to SEN and SGEN formation [66]. Additionally, postnatal deletion of the TSC1 gene in NSCs and their progenies in the SVZ causes SEN- and SEGA-like abnormalities in the lateral ventricles due to impaired neuroblast migration during neurogenesis [62]. Recent studies indicate that eNSCs lacking *TSC1* or *TSC2* genes rely on lipophagy to boost mTORC1 hyperactivation, driving tumorigenesis (Fig. 2) [66]. The metabolic changes in these deficient eNSCs could be targeted to correct aberrant differentiation. This suggests the TSC1-TSC2 complex has therapeutic potential for treating CTs, SENs, and SEGNs by regulating eNSCs and aNSCs through complex mechanisms.

### TSC1-TSC2 complex and germline stem cells

Gametes, the reproductive cells, are produced from germline stem cells (GSCs) throughout an individual’s fertile years. Unlike other stem cells, GSCs uniquely pass genetic information to future generations [67]. Niches support GSCs maintenance by balancing self-renewal and differentiation through external signals. The Drosophila ovarian GSC niche is a well-known model for studying GSC regulation in vivo. BMP signaling from cap cells in the GSC niche prevents GSC differentiation by repressing Bam transcription [68].

**Fig. 3:**
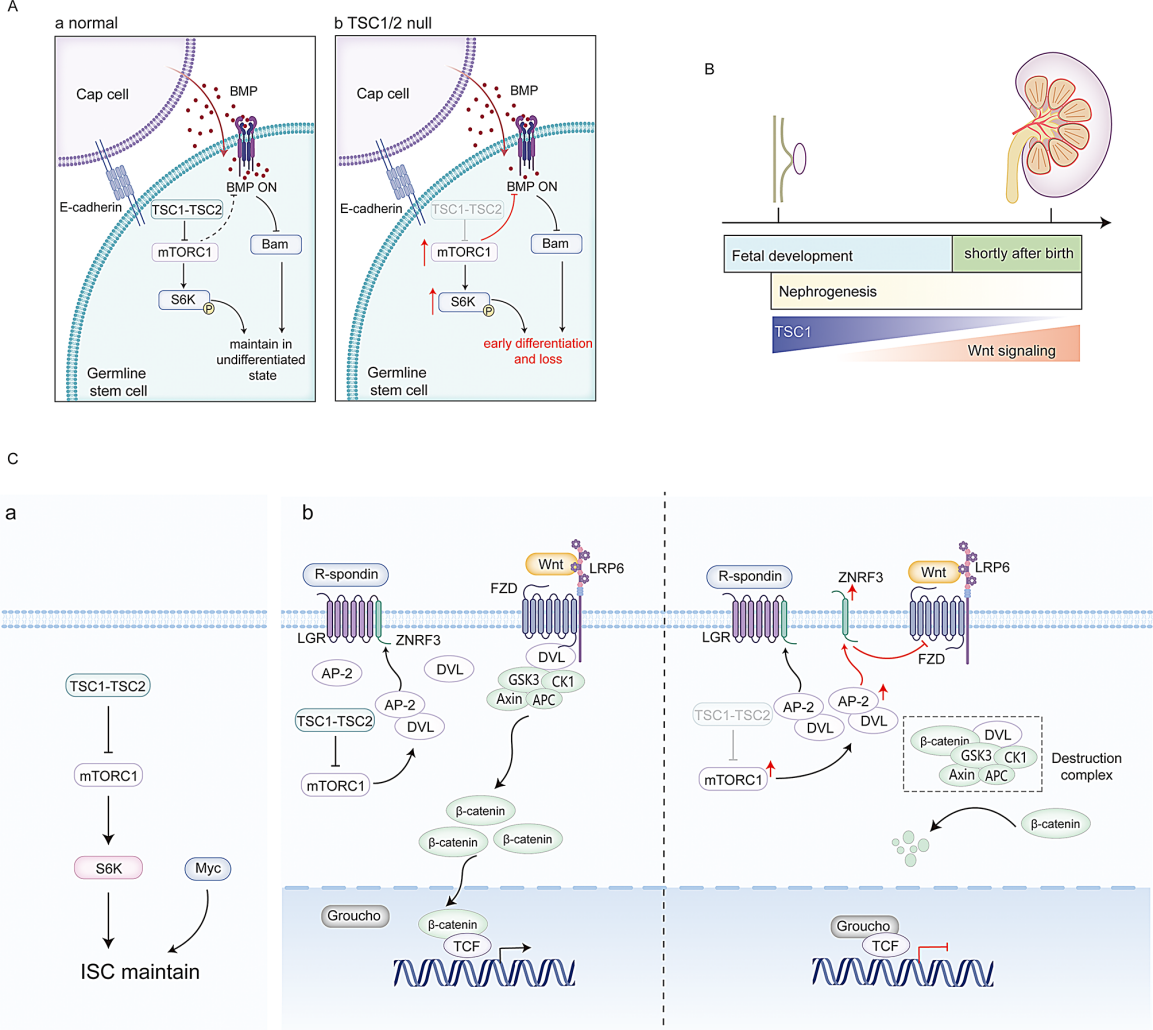
TSC1-TSC2 complex in ovarian GSCs, Nephron progenitor cells and ISCs. **(A).** TSC1-TSC2 complex in Drosophila ovarian GSCs. TSC1-TSC2 complex and BMP signal coordinate regulation of Drosophila ovarian GSCs maintenance. (**B).** TSC1-TSC2 complex in Nephron progenitor cells. TSC1-TSC2 complex regulates the altered sensitivity to the Wnt signaling pathway and regulates the proliferation of nephron progenitor cells. **(C).** TSC1-TSC2 complex in ISC. (**a**) TSC1-TSC2 complex regulate *Drosophila* ISCs maintenance by coordinating with Myc. (**b**) TSC1/2 regulates mouse ISC proliferation related to the Wnt/β-catenin signal

Loss of *TSC1* or *TSC2* genes in Drosophila ovarian GSCs leads to precocious differentiation and depletion, which can be reversed with the mTORC1 inhibitor rapamycin or by removing S6K [69]. In 2010, a research found that, in TSC1-mutated GSCs, BMP signaling decreases, but bam expression stays repressed. Mutations in the *TSC1* gene and bam lead to early differentiation into cystocytes (Fig. 3A) [69], highlighting the TSC1-TSC2 complex’s vital role in sustaining BMP signaling and preventing differentiation, independent of Bam [69]. This complex also influences Drosophila ovarian GSCs and their niches. Although the exact interaction mechanisms between TSC1/2-mTOR and BMP signaling remain unclear, these findings underscore importance of the TSC1-TSC2 complex in stem cell niches. GSCs across organisms share common mechanisms for germline survival, with TSC2 protein crucial for maintaining mouse spermatogonial progenitor cells (SPCs) [70]. Experiments show that SPCs prone to differentiation are less affected by TSC2 gene deletion than those with high self-renewal capacity. Thus, TSC2 is a key regulator of SPC fate, with the TSC2-mTOR pathway dynamically controlling SPC fate [70]. This study highlights stem cell heterogeneity and the need for more research on how the TSC1-TSC2 complex regulates their function.

### TSC1-TSC2 complex and nephron progenitor cells

The nephron is the kidney’s fundamental unit, crucial for electrolyte balance through filtration. Renal function hinges on the number of nephrons formed during nephrogenesis, which in mammals, depends on nephron progenitor cells at the ureteric bud (UB) tips [71–72]. Nephrogenesis occurs only during pregnancy or right after birth, ending when these progenitor cells are depleted (Fig. 3B). Understanding the fate of nephron progenitor cells is crucial for renal development. In mice, those with a *TSC1* gene deficiency show increased nephrogenesis and altered Wnt signaling sensitivity. Single-cell RNA sequencing reveals that TSC1^+/−^ cells translate more Wnt antagonists and fewer Wnt agonists (Fig. 3B). This study reveals how the TSC1 signaling pathway influences the Wnt pathway to control nephrogenesis [73]. TSC1^+/−^ nephron progenitor cells exhibit enhanced nephrogenesis without adverse effects, suggesting that *TSC1* gene editing could be therapeutically beneficial for conditions with reduced nephron function [74]. However, removing both *TSC1* gene alleles in these cells leads to fatal tubular lesions and cysts [75].

Renal diseases exhibit a high prevalence among individuals with tuberous sclerosis complex, often involving vascular smooth muscle tumors (AML) and cysts. This indicates TSC1 protein’s key role in nephron progenitor cell fate and its potential in regenerative medicine. To enhance its use in this field, it’s essential to analyze the factors causing different phenotypes in nephron progenitor cells with either a single or double *TSC1* gene deletion.

### TSC1-TSC2 complex and intestinal stem cells

Intestinal stem cells (ISCs) are residing the bases of crypts and sustain proliferation and differentiation to maintaining the intestinal homeostasis throughout life [76]. The intestine’s main roles are absorbing metabolites and providing environmental protection, functions believed to be supported by intestinal stem cells (ISCs) [76]. In Drosophila, ISCs give rise to enteroblasts (EBs) and then differentiate into enterocytes (ECs) or enteroendocrine cells (EECs), maintaining a balance between ECs and EEs. Recent research has significantly improved understanding of how the TSC1-TSC2 complex affects ISC fate determinations. For instance, deleting TSC1 or TSC2 genes in Drosophila ISCs disrupts proliferation and causes early differentiation into enterocytes (ECs) instead of enteroendocrine cells (EECs), regulated by Myc (Fig. 3C) [77]. In mice, TSC1 deletion in ISCs causes intestinal aging due to excessive proliferation, without altering differentiation [78]. The Wnt/β-catenin signaling pathway, vital for ISCs, is highly conserved evolutionarily. The TSC1-TSC2 complex regulates the Wnt/β-catenin pathway to control ISC proliferation. Dysfunction in this complex leads to mTORC1 overactivity, which enhances DVL and AP-2 interaction, suppressing Wnt/β-catenin signaling and impairing mouse ISC pluripotency (Fig. 3C). The exact mechanism of this interaction remains unclear [79]. Additionally, the Notch signaling pathway is crucial for maintaining intestinal homeostasis by regulating the differentiation of ISCs into absorptive and secretory cells in a specific ratio. This pathway regulates TSC2 protein expression, crucial for intestinal stem cell maintenance and differentiation [80]. The TSC1-TSC2 complex supports ISCs independently and with Wnt/β-catenin and Notch pathways. Understanding these interactions aids in advancing intestinal tissue regeneration and differentiation in regenerative medicine.

### TSC1-TSC2 complex and hematopoietic stem cells

**Fig. 4:**
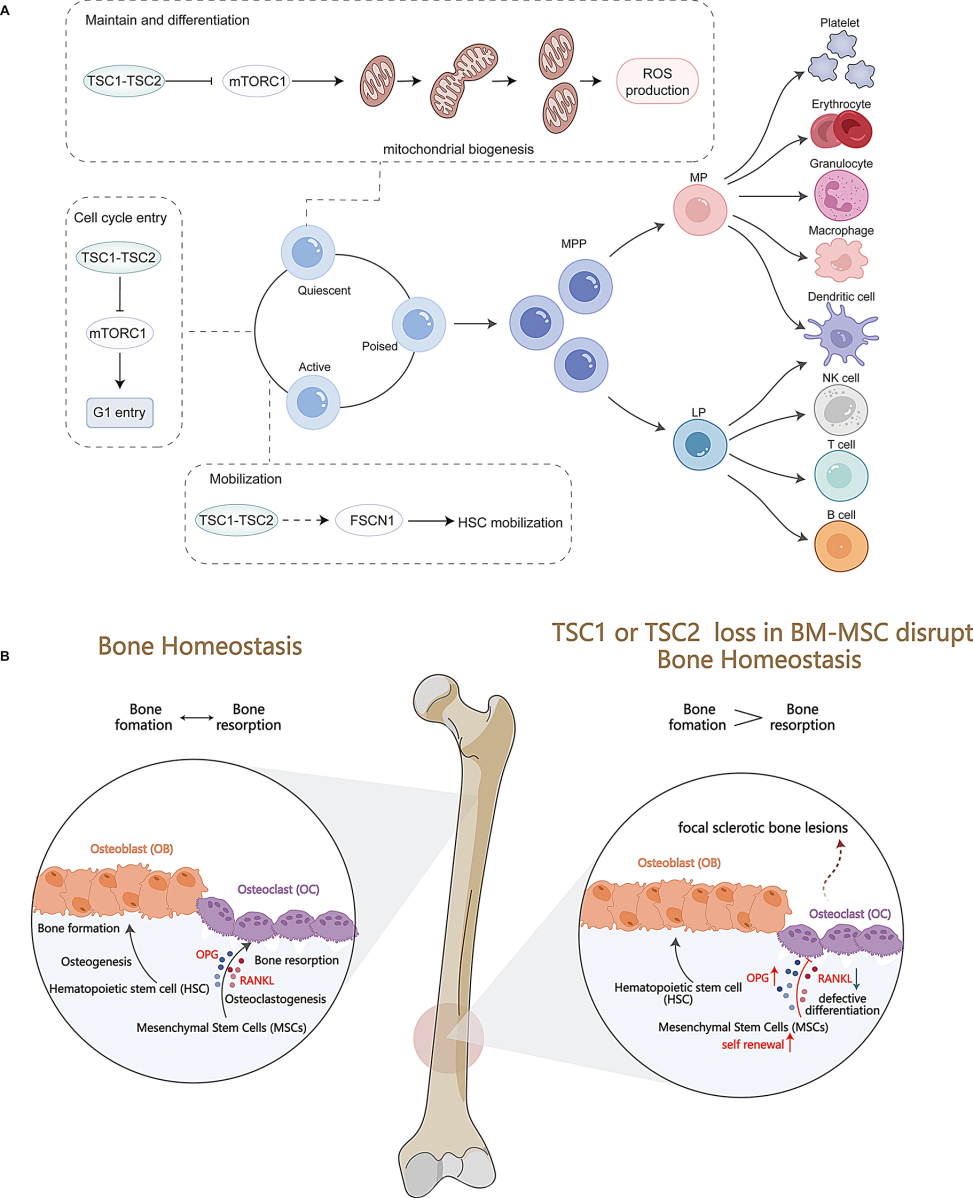
TSC1-TSC2 complex in HSCs and BM-MSCs. **(A).** TSC1-TSC2 complex in HSCs. TSC1-TSC2 complex regulates HSCs maintenance, differentiation, and mobilization in mTORC1-dependent way and mTORC1-independent way. **(B).** TSC1-TSC2 complex in BM-MSCs. TSC1-TSC2 complex regulates the level of OPG and RANKL, which BM-MSCs secretes. OPG and RANKL antagonistic regulate the proliferation of osteoclasts, maintaining bone homeostasis

Hematopoietic stem cells (HSCs) are rare, long-lasting stem cells that can self-renew and differentiate into all blood cell types, including red blood cells, megakaryocytes, myeloid cells (monocyte/macrophage and neutrophil), and lymphocytes [81]. They are crucial for the hematopoietic system’s roles in oxygen transport and immune defense. Proper regulation of HSC self-renewal and differentiation is essential for these functions. Deleting the *TSC1* gene in the mature hematopoietic system leads to increased HSC proliferation, mobilization, depletion, impaired long-term repopulation, and lineage development issues (Fig. 4A) [82]. These effects are confirmed in mice with TSC1 deletion in HSCs. TSC1 protein controls HSC self-renewal and differentiation through two mechanisms. The first mechanism involves the mTORC1 pathway, which keeps HSCs quiescent and functional by limiting mitochondrial biogenesis and lowering reactive oxygen species(Fig. 4A) [83]. The second mechanism, the FSCN1 pathway, regulates HSC mobilization (Fig. 4A) [82]. Additionally, 2-month-old mice lacking the *TSC1* gene in HSCs show aging-like traits, such as reduced lymphopoiesis and impaired hematopoietic reconstitution [82]. Understanding the TSC1 mechanism in HSCs could provide new insights into delaying senescence.

PTEN and TSC2 share similar biological functions in inhibiting tumor growth and regulating tissue and organism size. PTEN is a strong tumor suppressor that uses its phosphatase activity to negatively regulate the AKT pathway and positively influence TSC2. PTEN-deficient hematopoietic progenitors proliferate more abnormally than those lacking TSC2, resulting in increased AKT signaling with possible mTOR-independent effects [84]. Therefore, further research is needed to explore how these variations affect the regulation of hematopoietic stem cell fate.

### TSC1-TSC2 complex and mesenchymal stem/stromal cells

Mesenchymal stromal cells, first discovered in mouse bone marrow by Friedenstein, are a diverse group of cells including multipotent stem cells, progenitors, and differentiated cells [85–86]. They are found in the bone marrow and other tissue environments [86–87]. Caplan named them mesenchymal stem cells in 1990 due to their self-renewal and differentiation abilities [88]. These cells are promising for regenerative medicine because they can develop into various tissue-specific cell types, such as osteoblasts, adipocytes, and chondrocytes [89]. Deleting the *TSC1* gene in BM-MSCs activates the mTORC1 pathway, causing these cells to over-proliferate and not differentiate into osteoblasts (Fig. 4B), leading to shorter bones and less mineralization in mice [90]. Clinical data from tuberous sclerosis complex patients support these findings, highlighting the importance of the TSC1-TSC2 complex in regulating mTORC1 activity for BM-MSC maintenance and differentiation [90–91]. The TSC1-TSC2 complex’s regulation of mTORC1 is vital for BM-MSC maintenance and differentiation. In tuberous sclerosis complex patients, sclerotic bone lesions with excess minerals are a key clinical feature [8]. TSC1-deficient prx1 BM-MSC mice show worsening spinal and disc issues [92], likely due to disrupted MSC proliferation and differentiation, expanded MSC pools, and impaired differentiation into osteoblasts.

## Conclusion and perspectives

This review summarizes the regulatory role of TSC1-TSC2 complexes in stem cells and their disease implications. Over the past two decades, these complexes have been central to regulating the mTORC1-S6K/4EBP1 pathway, affecting global translation in various stem cell types [50]. The mTOR pathway also significantly influences energy metabolism. However, the effects of energy metabolism abnormalities in TSC1- or TSC2-deficient stem cells and their niche environments, crucial for stem cell maintenance, remain unclear. Senescence is strongly linked to dysfunctional mTOR signaling and metabolism [93]. In murine embryo fibroblasts, senescence results from TSC1/2 loss, which activates mTOR and disrupts PI3K- AKT signaling by downregulating PDGFR [94]. The TSC1-TSC2 complex is linked to the senescence of various stem cells, like hematopoietic and intestinal stem cells. Exploring the metabolic vulnerabilities related to this complex could offer new treatments for tuberous sclerosis complex. Additionally, TSC1-TSC2 influences stem cell behavior independently of mTORC1, aiding drug development for tuberous sclerosis complex.

In the study, TSC1 and TSC2 usually function as a protein complex to explore their role in stem cells. However, mutations in TSC1 or TSC2 lead to different clinical outcomes. Mutations in TSC2 are more common in individuals with intellectual disabilities and are linked to severe polycystic kidney disease [95]. Notably, renal progenitor cells with a heterozygous TSC1 deficiency show improved transplantation success and effective treatment of chronic kidney disease [74]. These results suggest that TSC1 and TSC2 may have different roles in organism development. Investigating the unique regulatory functions of TSC1 and TSC2 proteins in stem cells could advance personalized therapies and reveal beneficial roles in regenerative medicine.

Although rapamycin shows excellent therapeutic efficacy in TSC1/2-deficient animal stem cell models, it fails to improve TSC-related neural disorders like ASD in clinical trials. This is likely due to two main reasons: animal models do not capture the clinical complexity of tuberous sclerosis complex, and there is a lack of uniformity in developmental timelines across species. Human-induced pluripotent stem cells (iPSCs) provide a valuable system for studying tuberous sclerosis complex (TSC). Patient-derived iPSCs allow for the exploration of TSC’s complex clinical phenotype [96]. Additionally, a two-hit model of cortical tuber formation, developed in 2018 using CRISPR-Cas9 and iPSCs, aligns with TSC pathogenesis and aids in researching developmental heterogeneity over time [97]. Including hIPSC in TSC1/2 stem cell studies could aid clinical translation.

## Data Availability

All references are included in this review.

## Abbreviations

4E: BP eukaryotic initiation factor 4E­binding proteins
ADR1: Arrest-defective protein 1
AML: Vascular smooth muscle tumors
AMPK: 5′ AMP-activated protein kinase
ASD: Autism spectrum disorders
Bam: Bag-of-marbles
BM-MSCs: Bone marrow-derived mesenchymal stem cells
BMP: Bone morphogenetic protein
CC: Coiled-coil domains
CLIP: Caudal late interneuron progenitor
CNS: Central nervous system
CTs: Cortical tubers
DD: Dimerization domain
DG: Dentate gyrus
EB: Enteroblasts
EC: Enterocyte
EE: Enteroendocrine
eIF4E: Eukaryotic translation initiation factor 4E
eNSCs: Embryonic neural stem cells
ERM: Ezrin-radixin-moiesin
GAP: GTPase-activating protein
GSCs: Germline stem cells
HSCs: Hematopoietic stem cells
IPCs: Intermediate progenitor cells
iPSC: Induced pluripotent stem cell
IRS1: Insulin receptor substrate 1
ISCs: Intestinal stem cells
MCD: Malformation of cortical development
mLST8: Mammalian lethal protein with SEC13 protein 8
MSCs: Mesenchymal stem cells
mTOR: Mammalian target of rapamycin
mTORC1: Mammalian target of rapamycin complex1
NF-L: Neurofilament-light chain
NPCs: Neural progenitor cells
NSC: Neural stem cells
OB: Olfactory bulb
PKD1: Polycystic kidney disease 1
pNSCs: Postnatal neural stem cells
PTMs: Post-translational modifications
RGCs: Radial glial cells
RMS: Rostral migratory stream
S6K: Ribosomal S6 kinases
SEGNs: Subependymal giant cell astrocytomas
SENs: Subependymal nodules
SGZ: Subgranular zone
SPCs: Spermatogonial progenitor cells
SVZ: Subventricular zone
TBC1D7: Tre2-Bub2-Cdc16-1 domain family member 7
TFEB: Transcription factor EB
UB: Ureteric bud
